# Different sensitivity of germinal center B cell-like diffuse large B cell lymphoma cells towards ibrutinib treatment

**DOI:** 10.1186/1475-2867-14-32

**Published:** 2014-04-03

**Authors:** Xiaohui Zheng, Ning Ding, Yuqin Song, Lixia Feng, Jun Zhu

**Affiliations:** 1 Key laboratory of Carcinogenesis and Translational Research (Ministry of Education), Department of Lymphoma, Peking University Cancer Hospital & Institute, No.52 Fucheng Road, Haidian District, Beijing 100142, China

**Keywords:** Germinal center B cell-like diffuse large B cell lymphoma, Bruton’s tyrosine kinase, BCR, Apoptosis

## Abstract

**Background:**

Although rituximab in the combination of CHOP chemotherapy has been widely used as the standard treatment for several kinds of B-cell non-Hodgkin lymphoma (B-NHL), a great number of B-NHL patients treated with this immunotherapy still develop primary and secondary resistance. Recently Bruton’s tyrosine kinase (Btk) inhibitor ibrutinib showed promising therapeutic effect in relapsed/refractory CLL and B-cell NHL, which provided essential alternatives for these patients.

**Methods:**

The proliferation and apoptosis induction of tumor cells were measured by cell viability assay and Annexin-V staining. Western Blotting analysis and real-time PCR were used to detect the expression level of target proteins and chemokines production.

**Results:**

We demonstrated that ibrutinib inhibited the proliferation and induced apoptosis of GCB-DLBCL cell lines through suppression of BCR signaling pathway and activation of caspase-3. Furthermore, the chemokines CCL3 and CCL4 production from tumor cells were also found to be attenuated by ibrutinib treatment. But different cell lines exhibited distinct sensitivity after ibrutinib treatment. Interestingly, the decreasing level of p-ERK after ibrutinib treatment, but not the basal expression level of Btk, correlated with different drug sensitivity.

**Conclusions:**

Ibrutinib could be a potentially useful therapy for GCB-DLBCL and the decreasing level of p-ERK could become a useful biomarker to predict related therapeutic response.

## Introduction

Diffuse large B cell lymphoma (DLBCL), the most common subtype of non-Hodgkin lymphoma (NHL) with aggressive property could be divided into three subgroups based on gene expression profile: germinal centre B cell-like DLBCL (GCB-DLBCL), activated B cell-like DLBCL (ABC-DLBCL) and primary mediastinal B cell lymphoma [1,2]. R-CHOP, a combination of rituximab plus chemotherapy including cyclophosphamide, doxorubicin, vincristine and prednisolone, has been established as the first-line treatment for DLBCL, but approximately 30–40% of patients still become primary and secondary resistant to these drugs [3,4]. So there is an urgent demand for the novel target therapy, which could provide alternatives for the treatment of individuals with recurrent or refractory disease.

B cell antigen receptor (BCR) signaling pathway has been recognized essential for the development of normal B cell and pathogenesis of B cell malignancies [5-8]. Bruton tyrosine kinase (Btk), a crucial regulator within the BCR signaling pathway, belongs to non-receptor tyrosine kinase of Tec family that expressed in many hematopoietic lineages [9]. After antigen binding to BCR complex, Btk translocates from cytoplasm to membrane, clocking at IPI3 converted from IPI2 by PI3K [10,11]. After the phosphorylation at Tyr551 and Tyr223 residues, Btk activates phospholipase C gamma 2 (PLCγ2), which leads to Ca^2+^ mobilization and PKC activation [12,13]. PKC propagates downstream pathways such as nuclear factor κB (NF-κB) signaling and mitogen-activated protein (MAP) kinases, such as ERK1/2 that regulates cellular survival and apoptotic responses [14-17]. Thus, targeting small molecules within BCR signaling pathway, especially Btk inhibition would be a novel approach for treating B cell lymphomas.

In recent years, several novel agents targeting BCR signaling pathway, especially ibrutinib (PCI-32765), have shown great anti-lymphoma activities in preclinical study and clinical trials [18-20]. Ibrutinib is an irreversible and selective Btk inhibitor, which binds covalently to the target cysteine-481 residue [21]. In preclinical research, ibrutinib showed its cytotoxicity towards B cell malignancies, including chronic lymphocytic leukemia (CLL) and mantle cell lymphoma (MCL) by preventing Btk auto-phosphorylation [22,23]. Furthermore, 60% of patients with relapsed or refractory B cell malignancies achieved an objective response in a phase I open-label clinical trial [24].

The constitutive activation of NF-κB signaling sustained by chronic BCR pathway plays an essential role in proliferation of ABC-DLBCL cells, which had been demonstrated through shRNA interference experiment [25-27]. Although it is reported that the survival of GCB-DLBCL did not so much rely on activated NF-κB pathway [27], in our investigation we indeed found that the viability of some GCB-DLBCL cell lines was also inhibited by ibrutinib and different GCB-DLBCL cell lines showed diverse sensitivity.

## Results

### Ibrutinib inhibited the proliferation of GCB-DLBCL cell lines in a dose- and time-dependent manner

**Figure 1:**
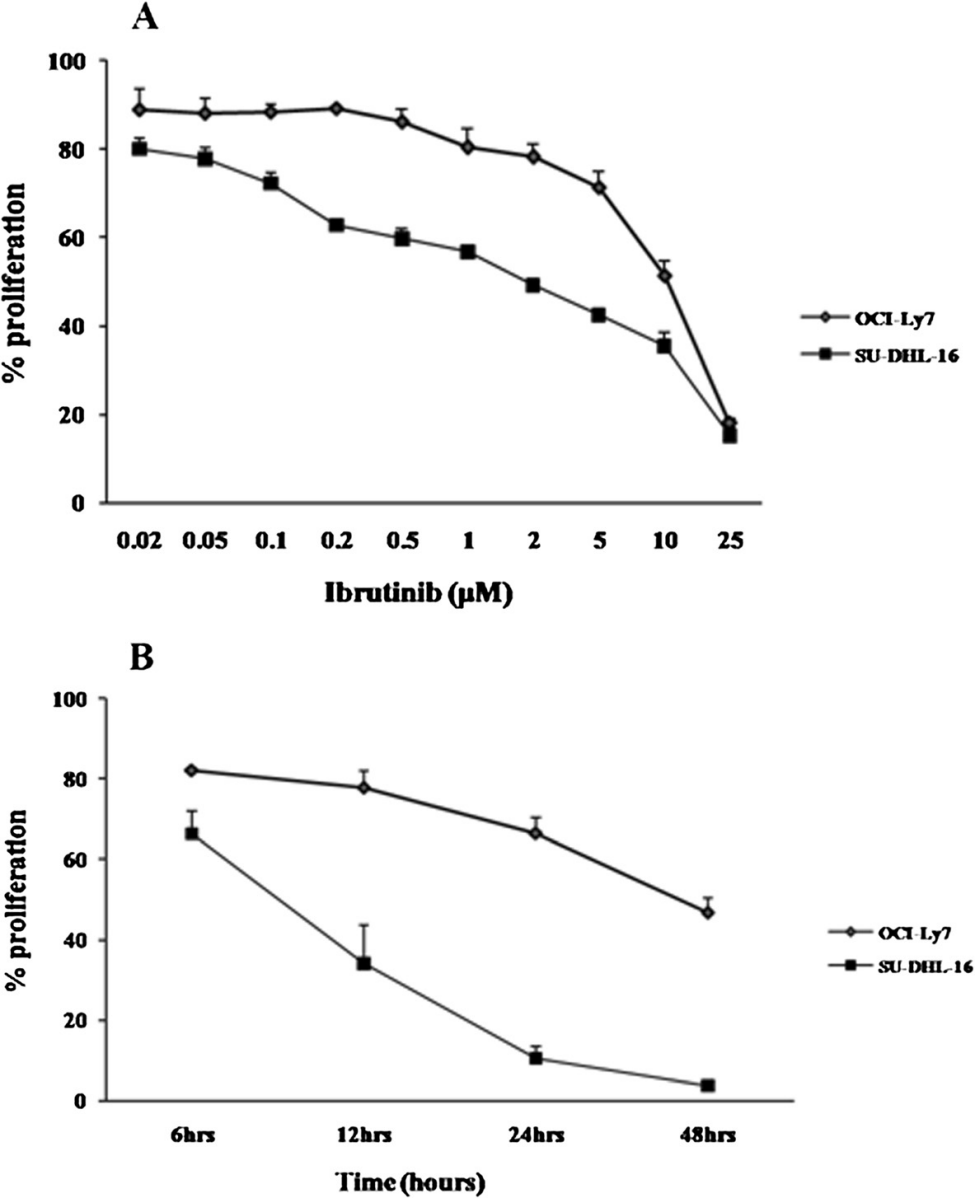
**Ibrutinib inhibited the proliferation of GCB-DLBCL cell lines in a dose- and time-dependent manner. (A)** SU-DHL-16 and OCI-LY7 cells (1 × 10^5^/ml) were treated with the increasing concentrations (0.02 to 25 μM) of ibrutinib or DMSO for 24 hours. **(B)** SU-DHL-16 and OCI-LY7 cells (1 × 10^5^/ml) were treated with10 μM of ibrutinib or DMSO for 6 to 48 hours. The viability of cells was determined using the Cell Titer-Glo luminescent cell viability assay as described in Materials and Methods. Results were expressed as the mean ± SD from triplicate cultures.

Firstly, we investigated the anti-tumor effects of ibrutinib in GCB-DLBCL cell lines SU-DHL-16 and OCI-Ly7. The cell viability assay demonstrated that the proliferation of tumor cells was inhibited by ibrutinib in a dose and time dependent manner (Figure 1A and B). But these two cell lines exhibited different drug sensitivity toward ibrutinib treatment. The IC_50_ values were 2.027 and 8.293 μM in SU-DHL-16 cells and OCI-Ly7 cells, respectively.

### Ibrutinib induced cell apoptosis in GCB-DLBCL cell lines by caspase dependent pathway

**Figure 2:**
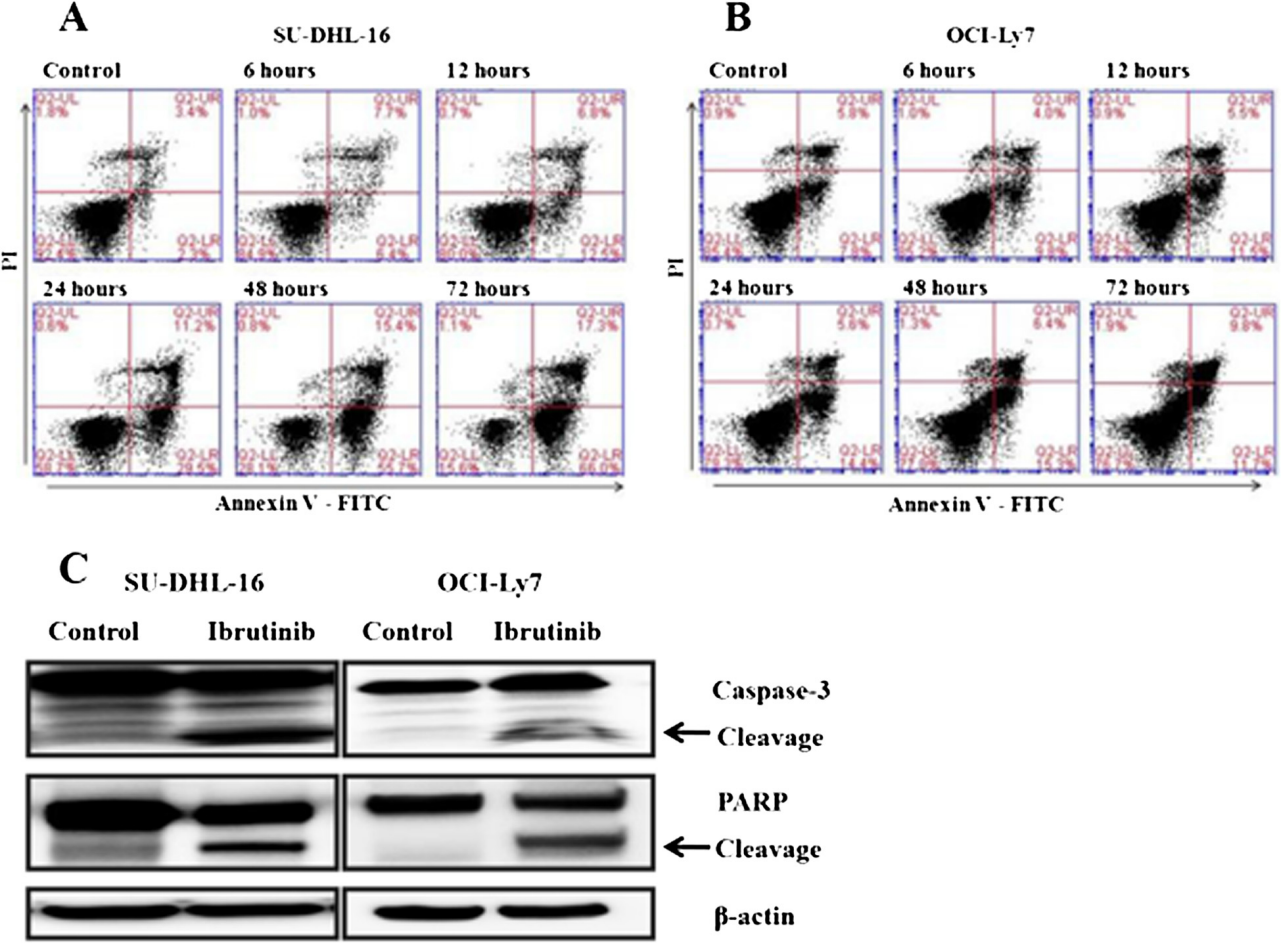
**Ibrutinib induced cell apoptosis in GCB-DLBCL cell lines by caspase dependent pathway. (A and B)** SU-DHL-16 and LY7 cells (1 × 10^5^/ml) were treated with a concentration of 10 μM of ibrutinib for 6–72 hours and Annexin-V and PI staining apoptotic cells were analyzed by flow cytometry. Cells in early stage apoptosis were defined as Annexin-V positive and PI negative and cells in late stage apoptosis were defined as Annexin-V and PI dual positive. **(C)** SU-DHL-16 and OCI-LY7 cells (1 × 10^5^/ml) were treated with10 μM of ibrutinib or DMSO for 24 hours. Then the total protein was prepared and the caspase 3 degradation and PARP cleavage were detected by Western Blot. β-actin was blotted as a loading control. Results were shown from one of three experiments and the representative results were shown in the figures.

To further investigate the mechanisms involved in anti-proliferation process by ibrutinib, Annexin-V and PI staining apoptotic cells were analyzed by flow cytometry. According to the previous results from cell viability assay and the dose of ibrutinib wildly used in inhibition of tumor growth in primary CLL cells [22], the concentration of 10 μM was used for subsequent experiments to demonstrate its anti-tumor activities in vitro. As shown in Figure 2A, SU-DHL-16 cells were tracked from Annexin-V and PI negative (no apoptosis) to Annexin-V positive and PI negative (early stage apoptosis) and finally to Annexin-V and PI dual positive (late stage apoptosis). The percentages of apoptotic cells in early and late stage were both gradually increased from 6 to 72 hours after ibrutinib incubation, which suggested that apoptosis induced by ibrutinib was time-dependent. But the apoptosis of OCI-Ly7 cells was not obviously induced by ibrutinib treatment (Figure 2B). It is reported that activation of caspase-3 and PARP is associated with the induction of apoptosis, therefore we detected the level of caspase-3 and PARP protein by Western Blot. As shown in Figure 2C, the caspase 3 and PARP cleavage were detected in both cell lines, which suggested an involvement of caspase pathway in ibrutinib induced apoptosis. But the density of caspase 3 and PARP cleavage in SU-DHL-16 cells was much higher than that in OCI-Ly7 cells, which confirmed the results from flow cytometry. This result also indicated that SU-DHL-16 cells were more sensitive to ibrutinib treatment than OCI-Ly7 cells.

### Ibrutinib down-regulated CCL3 and CCL4 gene expression in GCB-DLBCL cell lines

**Figure 3:**
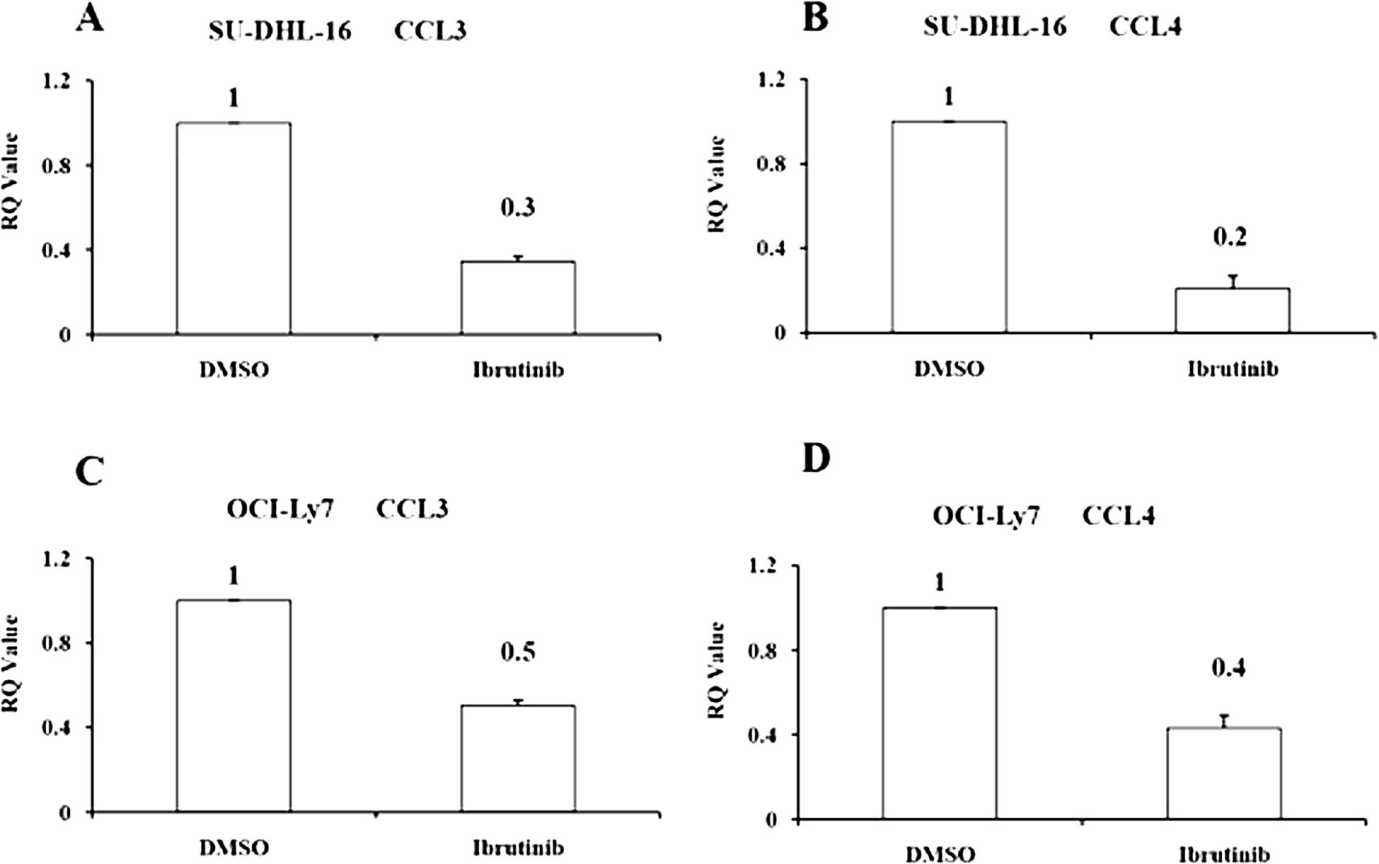
**Ibrutinib down-regulated CCL3 and CCL4 gene expression in GCB-DLBCL cell lines.** SU-DHL-16 and LY7 cells (1 × 10^5^/ml) were treated with a concentration of 10 μM of ibrutinib for 12 hours. Then total RNA was extracted by TRIzol reagent and performed a reverse transcription. RealTime-PCR analysis was done to determine the quantities of CCL3 and CCL4 mRNA. β-actin was used as the internal control gene for normalization. The expression levels of CCL3 and CCL4 of SU-DHL-16 cells after ibrutinib treatment were shown in Figure 3**A** and Figure 3**B**. The expression levels of CCL3 and CCL4 of OCI-Ly7 cells after ibrutinib treatment were shown in Figure 3**C** and Figure 3**D**. Results were expressed as the mean ± SD of three independent experiments. The differences in groups were assessed by Student’s t-test (*p* < 0.05).

It is reported that chemokines CCL3 and CCL4 secreted by B-CLL cells were highly regulated by BCR signaling pathway [28,29]. After the ibrutinib treatment, the mRNA expression of CCL3 and CCL4 from GCB-DLBCL cells were detected by Real-Time PCR. As shown in Figure 3, ibrutinib decreased the level of CCL3 and CCL4 gene expression in both cell lines, but the decreasing degree in SU-DHL-16 were much lower than that in OCI-Ly7 after ibrutinib treatment (*p* < 0.05).

### The basal level of Btk expression was not associated with different sensitivity to ibrutinib in GCB-DLBCL cell lines

**Figure 4:**
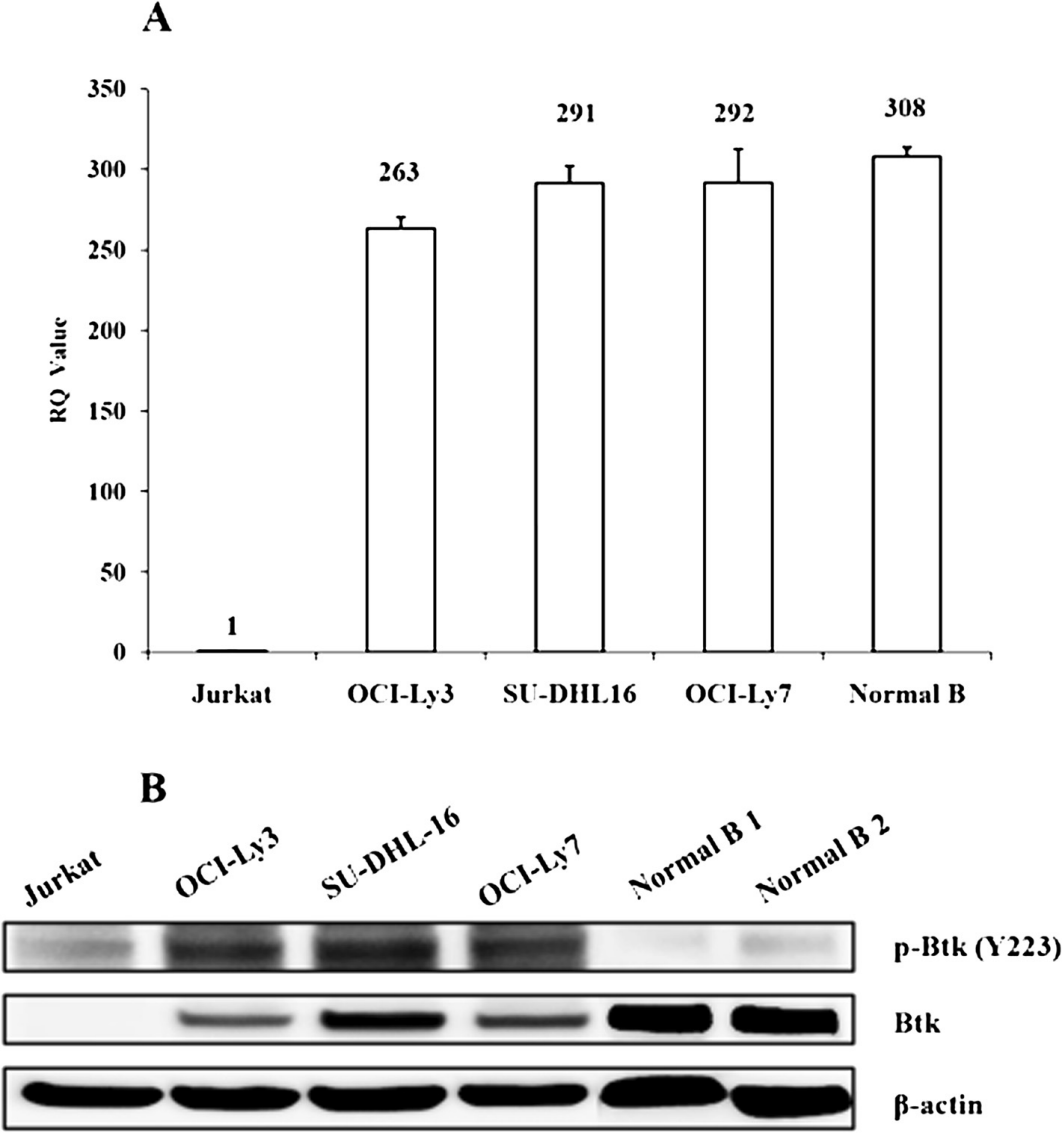
**The basal level of Btk expression was not associated with different sensitivity to ibrutinib in GCB-DLBCL cell lines. (A)** Total RNA from untreated Jurkat, OCI-Ly3, SU-DHL-16, LY7 and normal B cells were extracted by TRIzol reagent and performed a reverse transcription. RealTime-PCR analysis was done to determine the quantities of Btk mRNA. β-actin was used as the internal control gene for normalization. Results were expressed as the mean ± SD of three independent experiments. The differences in groups were assessed by Student’s t-test (*p* < 0.05). **(B)** Untreated Jurkat, OCI-Ly3, SU-DHL-16, LY7 and normal B cells were lysed using RIPA buffer for total protein. Western Blot was performed to detect the expression of p-Btk and Btk protein. β-actin was included as a loading control. Results were shown from one of three experiments and the representative results were shown in the figures.

Since ibrutinib exhibited distinct inhibitory activities in different GCB-DLBCL cell lines, next we sought to investigate the key regulator determined the response efficacy to Btk inhibition. Firstly, the expression of Btk in cells was examined at both mRNA and protein level. Compared with Jurkat cell line, three DLBCL cell lines had high level of Btk mRNA expression and the expression difference between GCB-DLBCL cell lines SU-DHL-16 and OCI-Ly7 was not statistically significant (Figure 4A). Phospho-Btk proteins were also observed at similar elevated levels in all three DLBCL cell lines (Figure 4B). The results revealed that the basal level and phosphorylation status of Btk expression were not associated with different sensitivity towards ibrutinib.

### Phosphorylation of ERK predicted the different response to ibrutinib

**Figure 5:**
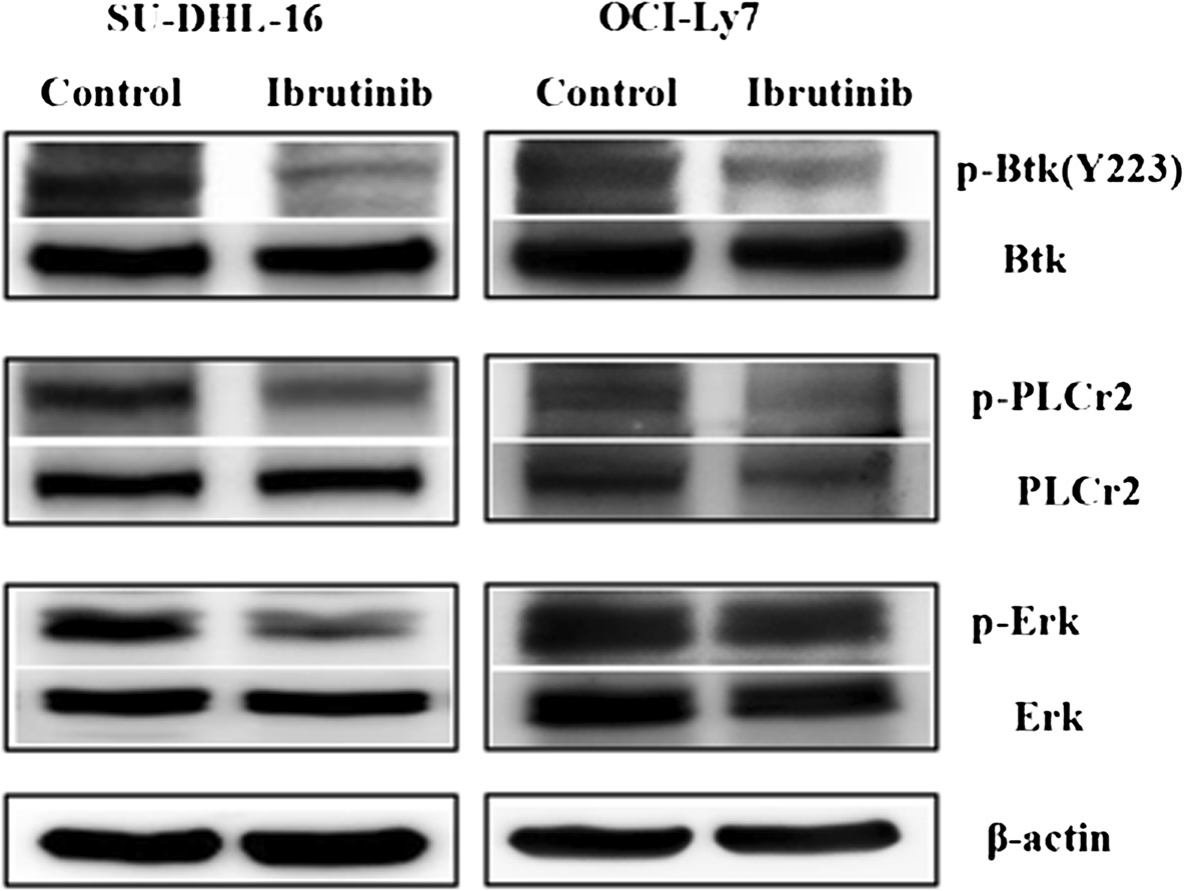
**Phosphorylation of ERK predicted the different response to ibrutinib.** Cells (1 × 10^5^/ml) were incubate with ibrutinib at a concentration of 10 μM for 24 hours, then lysed using RIPA buffer for the total protein. The expression of p-Btk, p-PLCγ2, p-ERK and Btk, PLCγ2, ERK protein was detected by Western Blot. β-actin was included as a loading control. Results were shown from one of three experiments and the representative results were shown in the figures.

Next we examined whether the different sensitivities between GCB-DLBCL cell lines attributed to the different inhibitory activities of activated BCR signal by ibrutinib. As shown in Figure 5, phosphorylation of Btk and PLCγ2 was similarly inhibited by ibrutinib in both sensitive cell line SU-DHL-16 and not-sensitive cell line OCI-Ly7, which suggested that ibrutinib induced the anti-lymphoma effect on GCB-DLBCL cell lines through the inhibition of BCR signal pathway. But phosphorylation of ERK was obviously inhibited by ibrutinib in SU-DHL-16 cells other than in OCI-Ly7 cells. These data demonstrated that inhibition of p-ERK, but not p-Btk and p-PLCγ2 determined the different sensitivity towards ibrutinib treatment.

## Discussion

In this experiment we investigated the inhibition effects induced by ibrutinib in GCB-DLBCL cells. Tumor cell proliferation was inhibited by ibrutinib in a dose- and time-dependent manner in both cell lines, but the IC_50_ value of OCI-LY7 cells was 4.4 times higher than that of SU-DHL-16 cells. SU-DHL-16 cells were more sensitive towards ibrutinib treatment than OCI-Ly7 cells.

Many investigations have shown that the targeted inhibitors of crucial tyrosine kinases in BCR signaling pathway, such as Syk inhibitor PRT060318 and the inhibitor of Src family kinases (SFKs) dasatinib, could prevent the proliferation of GCB-DLBCL cell lines by causing cell-cycle arrest or inducing cell apoptosis [30,31]. Our results above also demonstrated that ibrutinib, as an important inhibitor of BCR signal activation and transduction, inhibited proliferation of tumor cells in a dose and time dependent manner.

Herman et al. has demonstrated that ibrutinib could induce apoptosis in CLL cells through caspase-dependent pathway [22]. Similarly, we also found that ibrutinib could induce a time-dependent apoptosis with the degradation of caspase-3 and cleavage of PARP in SU-DHL-16 but not so much in OCI-Ly7 cells, which confirmed the distinct sensitivity towards ibrutinib treatment between different GCB-DLBCL cell lines.

It is reported that the secretory chemokines CCL3 and CCL4 played an important role in the cross talk between CLL cells and their microenvironment, which promoted the proliferation and metastasis of tumor cells [28,29]. Ponader et al. demonstrated that ibrutinib down-regulated the secretion of chemokines CCL3 and CCL4 in CLL cells both in vitro and in vivo [32]. In our experiment, we also observed that the expression of CCL3 and CCL4 were dramatically decreased after treatment of ibrutinib, but the decreasing degree were much more obviously observed in SU-DHL-16 than that in OCI-Ly7 (*p* < 0.05).

Many reported demonstrated that the activation status of downstream kinases related to outcome of the target inhibitor. Yang et al. found that dasatinib induced considerable reduction in the phosphorylation of Syk and PLCγ2 in sensitive cell lines, other than in resistance cell lines [30]. Phosphorylated-Syk and PLCγ2 might serve as potential biomarkers to predict response to dasatinib treatment. In much the same way, Cheng et al. also found that phosphorylation of PLCγ2 and AKT was reduced in a dose-dependent fashion in sensitive but not resistant cell lines after Syk inhibitor PRT060318 treatment [31]. Thus, to further investigate the possible mechanisms of different sensitivities towards ibrutinib treatment, the basal level and phosphorylation status of key regulatory enzymes (Btk, PLCγ2 and Erk1/2) of BCR signal pathway were analyzed by western blotting. We examined the phosphorylation of Btk and downstream molecular PLCγ2 and ERK in GCB-DLBCL cells lines before and after ibrutinib treatment. Phosphorylation of Btk and PLCγ2 was similarly inhibited by ibrutinib in both cell lines, but phosphorylation of ERK was dramatically inhibited in SU-DHL-16 cells but not in OCI-Ly7 cells. Therefore, inhibitory level of p-ERK could be an important response predictor to ibrutinib. On the other hand, the distinct genetic background of SU-DHL-16 and OCI-LY7 cells may also contribute to the different sensitivity toward ibrutinib treatment, but the further experiments are needed to investigate the possible mechanisms in detail.

Together, in this study we revealed that ibrutinib inhibited the proliferation of GCB-DLBCL cell lines through down-regulation of BCR signaling pathway and activation of caspase-3. Phosphorylation level of ERK in GCB-DLBCL could be a useful response predictor to ibrutinib.

## Materials and methods

### Reagents and antibodies

Ibrutinib was purchased from Selleck Chemicals (Houston, TX, USA. Cat No. S2680) and dissolved to a 40 mM stock solution in dimethylsulfoxide (DMSO). Antibodies for phospho-Tyr223-Btk (5082), phospho-ERK1/2 (4370), phospho-Tyr759-PLCγ2 (3874), Btk (8547), ERK1/2 (9102), PLCγ2 (3872) and Caspase-3 (9662) used in western blotting were all purchased from Cell Signaling Technology (Danvers, MA, USA). Anti-PARP antibody was obtained from BD Pharmigen (Cat No.556362, BD Pharmigen, San Jose, California, USA). β-actin used as an internal control for western blotting was generated by Sigma-Aldrich (Cat No. A5441, Sigma-Aldrich, St. Louis, MO, USA). HRP-conjugated anti-rabbit IgG (Cat No.458) and HRP-conjugated anti-mouse IgG (Cat No.330) were purchased from MBL (MBL International Corporation, Nagoya, Aichi-ken, Japan).

### Cell lines and culture conditions

The non-Hodgkin lymphoma cell lines SU-DHL-16, OCI-Ly3 and OCI-Ly7 were obtained from Professor Fu Kai in University of Nebraska Medical Center. The SU-DHL-16 and OCI-Ly7 cell lines possess t(14;18)(q32;q21) and t(8;14)(q24;q32) translocation, respectively. These cell lines were cultured in Dulbecco’s modified Eagle’s medium (DMEM; Invitrogen, Life Technologies, Carlsbad, CA, USA) supplemented with 10% fetal bovine serum (FBS; Gibco, Life Technologies), L-glutamine and penicillin–streptomycin in humidified 5% CO_2_ at 37°C.

### Cell viability assay

In vitro cytotoxicity assays were performed by Cell Titer-Glo Luminescent Cell Viability Assay (Cat No. G7572, Promega Corporation, Madison, WI, USA). Cell lines were plated in opaque-walled 96-well plates at a density of 1 × 10^5^/ml and incubated with various concentrations of ibrutinib for 24 hours. Then, 20 μL of the assay reagent was added to each well, and the cell lysates were incubated on an orbital shaker at room temperature for 10 minutes. Luminescent signal was measured by LMax II (Molecular Devices, Sunnyvale, CA, USA). The half maximal inhibitory concentration (IC 50) of ibrutinib was calculated using Graphpad prism 5 software (GraphPad Software Inc., San Diego, CA, USA).

### Western blotting

Cell lines treated or non-treated with ibrutinib were harvested and lysed using RIPA buffer (Cat No. 9806, Cell Signaling Technology, Danvers, MA, USA) supplemented with protease inhibitor cocktail tablets (Cat No. 04693124001, Roche, Mannheim, Baden-Wuerttemberg, Germany) to get the protein in total cell extracts. Then equivalent amounts of protein were separated by sodium dodecyl sulfate polyacrylamide gel electrophoresis (SDS-PAGE) on which separating gel contained 4-15% acrylamide (Cat No. 456–1083, Bio-Rad, Hercules, CA, USA), transferred to polyvinylidene fluoride (PVDF) membrane (Cat No. IPVH00010, Millipore Corporation, Billerica, MA, USA) and probed with appropriate primary and secondary antibodies mentioned above to study the BCR signaling pathway and apoptosis-related protein. ECL select western blotting detection reagent (Cat No. RPN 2235, GE Healthcare, Little Chalfront, Bukinghamshire, UK) was used for detection on a Fluor Chem HD2 (Cell Biosciences, Santa Clara, CA, USA).

### Quantification of apoptosis

After incubated with ibrutinib or not for the indicated periods, 1 × 10^6^ cells were washed with cold PBS, labeled using Annexin-V-FITC and propidiumiodide (PI) in binding buffer according to the manufacturer’s protocol of the Annexin-V-FITC apoptosis detection kit purchased from Beijing Biosea Biotechnology (Cat No. CX1001, Beijing, China) and analyzed by BD Accuri C6 flow cytometer (BD Biosciences, San Jose, California, USA). Early stage apoptosis cells were defined as Annexin-V positive and PI negative and late stage apoptosis cells were defined as Annexin-V and PI dual positive.

### RNA extraction and real-time PCR

Total RNA was extracted using TRIzol reagent (Cat No. 15596–026, Life Technologies, Carlsbad, CA, USA), according to the manufacturer’s instructions. Reverse transcription was performed using TransScript First-Strand cDNA Synthesis SuperMix (Cat No. AT301, TransGen Biotech, Beijing, China). Real-Time PCR was performed with the specially designed primers and probes and Platinum Quantitative PCR SuerMix-UDG (Cat No.11730-017, Life Technologies, Carlsbad, CA, USA). The primers and probes were as follows: Btk sense CCGGAAGACAAAAAAGCCTCTT, antisense GGCGGTAGTGGCTTTTTCAA, Probe FAM-CCCAACGCCTGAGGAGGACCAGA-TAMRA; CCL3 sense GAGCCCACATTCCGTCACCT, antisense CACTGGCTGCTCGTCTCAAA, Probe FAM-CCACTGCTGCCCTTGCTGTCC-TAMRA; CCL4 sense CAGCGCTCTCAGCACCAA, antisense AGCTTCCTCGCAGTGTAAGAAAA, Probe FAM-CTCAGACCCTCCCACCGCCTGC-TAMRA; ACTB sense CCTGGCACCCAGCACAAT, antisense GCCGATCCACACGGAGTACT, Probe FAM-ATCAAGATCATTGCTCCTCCTGAGCGC-TAMRA. After UDG incubation (50°C for 2 min) and pre-amplification (95°C for 2 min), the PCR was amplified for 40 cycles (95°C for 15 s and 60°C for 30 s) on a ABI 7500 Real Time PCR System (Life Technologies, Carlsbad, CA, USA). The fold change in mRNA was calculated by the 2^-ΔΔCt^ method.

### Preparation of human peripheral blood B cells

Peripheral blood lymphocytes were isolated from 15 ml EDTA-blood obtained from healthy volunteers using Lymphocyte Separation Medium (Cat No. LTS1077, Tian Jin Hao Yang Biological Manufacture CO.,LTD, Tianjin, China) according to the protocol from the manufacturer. Normal B cells were separated from the peripheral blood lymphocytes using B Cell Isolation Kit (B-CLL) human (Cat No. 130-093-660, Miltenyi Biotec, Bergisch Gladbach, Germany) according to the protocol from the manufacturer. This research protocol was approved by our Institutional Review Board.

### Statistical analysis

Statistical significance from controls was assessed by Student’s t-test and *p* < 0.05 values were accepted as statistically significant. Results represent the mean ± standard deviation (SD) of three independent experiments. Western blotting experiments were repeated three times or more and the representative results were shown in the figures.

## Competing interests

All authors declare that they have no competing interests.

## Authors’ contributions

ZJ, SYQ and DN designed the study and review the final manuscript. ZXH designed, performed the experiment, analyzed data and wrote the manuscript. FLX helped to preparation of human peripheral blood B cells. All authors read and approved the final manuscript.
